# Preliminary study on Se-enriched *Lentinula edodes* mycelium as a proposal of new feed additive in selenium deficiency

**DOI:** 10.1371/journal.pone.0233456

**Published:** 2020-05-21

**Authors:** Bożena Muszyńska, Ewelina Szacawa, Dorota Bederska-Łojewska, Katarzyna Dudek, Bartosz Pomierny, Anna Włodarczyk, Katarzyna Kała, Jan Lazur, Piotr Suchocki, Bogusława Budziszewska, Dariusz Bednarek, Marek Pieszka

**Affiliations:** 1 Department of Pharmaceutical Botany, Faculty of Pharmacy, Jagiellonian University Medical College, Krakow, Poland; 2 Department of Cattle and Sheep Diseases, National Veterinary Research Institute, Pulawy, Poland; 3 Department of Animal Nutrition and Feed Science, National Research Institute of Animal Production, Balice, Poland; 4 Department of Biochemical Toxicology, Faculty of Pharmacy, Jagiellonian University Medical College, Krakow, Poland; 5 Department of Bioanalysis and Drug Analysis, Faculty of Pharmacy, Warsaw Medical University, Warszawa, Poland; Chinese Academy of Sciences, CHINA

## Abstract

The presence of selenium in European soil is low and this causes its deficiency in livestock and, in consequence, in humans. This study aimed to obtain *Lentinula* (*L*.) *edodes* mycelium with the maximum content of selenium. This species was used for experiment based on its documented medicinal properties. Calves were fed with selenium-enriched *L*. *edode*s mycelium, and serum selenium concentration, average daily weight gains and selected immune parameters were estimated. The selenium-enriched mushroom was found to be safe based on cytotoxicity tests (MTT and LDH tests) and for this reason it was used for further experiments. The mean quantity of selenium in the serum of calves fed with selenium-enriched *L*. *edodes* mycelium was significantly higher than that of control calves. Additionally, the calves fed with selenium-enriched *L*. *edodes* mycelium had higher body weight gains than those of control calves. White blood cell counts and subpopulations of lymphocytes in the experimental and control calves were within the reference range. The administration of *L*. *edodes* enriched with selenium had a beneficial effect on state of health of the calves.

## Introduction

The soil of Central European countries is deficient in selenium (Se), which is an essential element for livestock and humans. Selenium-deficient soil translates into its deficiency in livestock, and in turn into its deficiency in humans. This deficiency has been linked to the increased incidence of serious diseases such as cancer and muscular weakness. With this awareness, Europeans are becoming more concerned about finding the possible solution to this deficiency [1].

The 1960s saw the first diagnoses of nutritional muscular dystrophy in calves, the cause of which was selenium deficiency. Deficiency of selenium leads to economic losses such as reduced milk yield, fertility problems, mastitis, and metritis in cattle [2, 3]. In the same decade, selenium was known to be an element essential for life, indispensable for the maintenance of the normal functioning of the organism and its proper development. It is a part of amino acids such as selenocysteine which are the structural elements of several proteins and are significant for homeostasis. Selenium is a component of glutathione peroxidase, which limits the harmful processes of lipid and nucleic acid peroxidation, thus protecting the cells from deformation and genetic damage. It shows preventive activity related to cancer of the colon, lungs, larynx, prostate, stomach, and esophagus. In addition it stimulates the immune system, thus exhibiting anti-inflammatory and antiviral properties (it inhibits the progression of HIV infection, limiting the development of AIDS). All deviations from the normal status level of selenium of an in the mammals organism are associated with an increase in the risk of the occurrence or progression of serious diseases [4]. Selenium is an important micronutrient in animals, but there is a narrow margin between the adequate and potentially toxic concentrations in the diet. Presented studies have demonstrated that supplementation of selenium-deficient cows has a positive effect on their fertility and growth, and stimulates their immune system [5, 6, 7]. Previous analysis has mainly focused on comparing doses, forms, and the digestibility of selenium additives. The selenium content in the body depends primarily on its dietary supply and chemical form and the presence of other compounds or elements that may interfere with its absorption. The most commonly used inorganic forms of selenium are sodium selenite (Na_2_SeO_3_) and sodium selenate (Na_2_SeO_4_), whereas the equivalent organic form is primarily selenomethionine (Se-Met), which occurs in yeast. Selenium is in the fruiting bodies of edible mushrooms, which generally are the perfect organisms to be used for the preparation of medicated supplements due to their high capacity to accumulate elements and their numerous therapeutic effects (e.g. immunostimulatory, anti-inflammatory, and prebiotic). [8, 9, 10, 11].Elements including selenium occur in the fruiting bodies of 200 species of edible mushrooms belonging to 21 families. *Boletus edulis* in the Boletaceae family contains 20 μg Se/g d.w. (dry weight), Agaricaceae mushrooms in the natural state contain 5 μg Se/g d.w., and the remaining species of mushrooms contain no less than 1 μg Se/g d.w. [11, 12]

*Lentinula* (*L*.) *edodes* (Berk.) Pegler (Basidiomycota) is a particularly interesting species. Medicines based on *L*. *edodes* are used to support cancer therapy due to their immunostimulatory and antioxidative effect [13, 14, 15]. Similarly to other species of mushroom, the fruiting bodies of *L*. *edodes* constitute a good source of vitamins and can accumulate selenium [16, 17, 18].

Developing an appropriate combination of selenium and mycelium to deliver the proper amount of selenium to the consumer constitutes a highly important challenge. Therefore, in this study, we aimed to optimize the conditions for an *in vitro* culture to obtain mycelium with the maximum Se(IV) content. For this purpose, mycelia from liquid cultures were used due to the biotechnological possibilities of enriching culture media with the selenium form of selenitetriglycerides. In addition, use of liquid cultures enabled mycelium to be obtained in a controlled manner and under standardized conditions, which afforded reproducibility and repeatability [19, 20]. *L*. *edodes* was chosen as a very suitable mycelium because of its unique medical properties.

To determine the safety of selenium-enriched mycelium in the living organisms, we conducted cytotoxicity tests (MTT– 3-(4,5-dimethylthiazol-2-yl)-2,5-diphenyltetrazolium bromide assay and LDH–lactate dehydrogenase assay) using the SH-SY5Y human neuroblast cell line.

The objective of this study was to determine the effect of Se-enriched *L*. *edodes* on serum selenium concentration, nonspecific immune response (white blood cell counts and the percentage of lymphocyte subpopulations) and average daily gains (ADG) of calves. The results of our study showed that the developed material containing the mycelium of *L*. *edodes* enriched with selenium can be used to supplement deficiencies of this element in livestock. The product is intended for use as a feed additive for calves.

## Materials and methods

### Reagents

Methanol was of Suprapur^®^ grade and was obtained from Merck (Darmstadt, Germany). For the determination of Se(IV), we used Ni(NO_3_)_2_ as the chemical modifier (Fluka, Seelze, Germany); HNO_3_ and standard solutions which contained 100 ng/mL of Se(IV) were obtained from Sigma-Aldrich Chemical Company (Darmstadt, Germany). Double-distilled and deionized water was prepared using the EASYpure RF compact ultrapure water system (Barnstead/APS Water Services, Lake Balboa, CA, USA). A mixture of selenitetriglycerides was synthesized by the reaction of Se(IV) with sunflower oil. The synthesis was performed at the Department of Bioanalysis and Drug Analysis of the Medical University of Warsaw [19].

### Mushroom material and mycelium preparation

In this study, we used fresh fruiting bodies of *L*. *edodes* (Berk.) Pegler (commonly known as the shiitake mushroom) of commercial origin as the study material. Representative samples of mushrooms were delivered to the Department of Pharmaceutical Botany of the Jagiellonian University Medical College. The taxonomic identification of fruiting bodies was performed using MycoKey 4.1 (http://www.mycokey.com). The explants originating from the young fruiting bodies were degreased with 70% ethyl alcohol for 15 s and then sterilized for 2–5 min in 15% hypochlorous acid. After triple rinsing with double-distilled water, the sterile fruiting body fragments were transferred under laminar airflow onto Oddoux medium solidified with agar [21]. Subsequently, to obtain the maximum growth of the mycelium, the biomass from the solid medium was transferred into a modified liquid medium in Erlenmeyer flasks (500 mL) containing 250 mL of the medium and a baseline inoculum of 0.1 g was added to the medium. The cultures were incubated at a temperature of 25±2°C, in a photoperiod of 16 h of 900 lx light and 8 h of darkness, and with constant shaking at 140 rpm on an ALTEL shaker (Poland) for 10 days.

### Production of *L*. *edodes* mycelium enriched with Se in biofermenter

*L*. *edodes* biomass was obtained on liquid Oddoux medium enriched with Se(IV) in the form of selenitetriglycerides [19]. The culture medium was supplemented with 25 and 50 mg/L of Se(IV) (0.5 and 1 mL of selenitetriglicerides per liter of the medium), respectively.

To obtain efficient and significant growth of biomass, the mycelium from Erlenmeyer flasks was transferred to a biofermenter, in which the volume of the medium was 10 L; the cultures were mixed by the inflow of air (air flow rate: 2100 L/h) passed through the sterile antibacterial filters. Carbon dioxide formed during the growth of the mycelium was constantly removed. The cultures were grown at a temperature of 25±2°C and under the natural photoperiod. After 10 days of growth in the biofermenter, the mycelium was separated from the medium and was frozen and lyophilized (Freezone 4.5 lyophilizer, Labconco; temperature:–40°C).

### Selenium determination

The determination of total content of Se was by atomic absorption spectroscopy (AAS) with the use of an Avanta Ultra Z spectrometer (GBC, Dandenong, Australia).

The samples were mineralized by exactly 0.1 g of mycelia and medium lyophilizate being measured into a teflon crucible, 3 mL of 65% HNO_3_ being added and the solution being processed in a microwave mineralizer. The process of mineralization was conducted according to the manufacturer’s instructions (Plazmatronika microwave mineralizer, model BM-1S).

Preparation of sample solutions started with the transfer of the mineralized solutions from the previous step into a 25 mL volumetric flask and adjustment with water. Next, 0.05 mL of this solution was placed in a 10 mL volumetric flask and adjusted with 1% HNO_3_ solution containing 5 mg of Ni(NO_3_)_2_ in 1 mL. This mixture was used as a sample to conduct measurements with the ASA method.

Preparation of standard solution was carried out using a standard solution of 100 ng of Se(IV)/mL in 1% HNO_3_ solution containing 5 mg Ni(NO_3_)_2_ in 1 mL was used as a solvent. A calibration curve was created automatically by placing 10 μl of mixtures containing 0; 10; 40 and 100 ng of Se(IV)/mL in a graphite furnace. The concentration of the standard solution was 100 ng of Se(IV)/mL. Every measurement was repeated 3 times. Quality of the performed analyses was tested using the certified reference material BCR-185R (Sigma-Aldrich). All the results were in agreement with the certified value of the selenium concentrations.

Relative Standard Deviation (RSD) measurements should not exceed 10%. The final measuring dilution of the analyzed solution varied and depended on the Se content in the sample. The samples were diluted in such a way that the result read by the apparatus was within the range of 30 to 100 ng Se (IV)/mL (Table 1).

**Table 1:** Program of graphite furnace in the spectrometer.

Stage	Temp. [°C]	Time of temp. rise [°C/s]	Time of temp. maintained [s]	Air flow [l/min]
Drying	110	10	10	3.0
„	130	1.0	10	3.0
Ashing	400	10	10	3.0
„	600	10	2	0.0
„	400	1.0	1.0	0.0
Atomization	2200	0.5	0.5	0.0
Cleaning	2300	0.1	0.9	3.0

### Cytotoxicity test

Lyophilized mycelia from liquid cultures with and without selenitetriglyceride were extracted three times with 100 mL of methanol using a Sonic-2 sonicator bath at a frequency of 49 kHz for 30 min in 25°C (Polsonic, Warszawa, Poland). Then, the extracts were evaporated to dryness in an evaporator at 40°C under pressure (200 mBa; Büchi, Germany). To verify the safety of selenium-enriched mycelium in living organisms, we performed cytotoxicity tests of the obtained extracts using LDH and MTT assays and SH-SY5Y cell line [22].

### Cell culture

The SH-SY5Y neuroblastoma cell line was obtained from the ATCC (American Type Culture Corporation). Briefly, the cells were cultured in Dulbecco’s modified Eagle’s medium (Gibco-BRL, Eggenstein, Germany) supplemented with 10% fetal bovine serum (FBS) (Gibco-BRL) and containing 100 units/mL of penicillin and 100 mg/mL of streptomycin (Sigma Co, St. Louis, MO, USA). The cells were incubated in a humidified atmosphere of 5% CO_2_/95% O_2_ at 37°C. After reaching 80% confluency, the cells were harvested and seeded at a density of 5 × 10^5^ cells per well in 96-well plates for the determination of LDH release, and for the determination of MTT reduction, the cells were seeded at a density of 8 × 10^5^ cells per well in 96-well plates. SH-SY5Y is a reliable model that is suitable for the evaluation of neurotoxicity and neuroprotection of drugs and other compounds [23]. It is a human-origin cell line with dopaminergic phenotype and is commonly used as an *in vitro* model in various research regarding neurodegenerative disorders, neurotoxicity, and oxidative stress. These characteristics indicated it to be appropriate for use in this study.

### Cell culture treatment

The extracts were prepared by dissolving mushroom material (2 mg) in 1 mL of ethanol:double distilled water mixture (v/v, 1:10). SH-SY5Y cells were treated with these extracts at a concentration of 25 μg/mL. Then, 48 h later, H_2_O_2_ (0.5 mM) was added and the cells were cultured for the next 24 h. The control cultures were supplemented with appropriate amounts of vehicle (ethanol:ddH_2_O, 1:10). After the incubation time cells were subjected to LDH and MTT assays.

### Measurement of LDH release

The H_2_O_2_-induced cytotoxicity in SH-SY5Y cells was quantified by measuring the efflux of LDH into the culture media 24 h after the treatment with H_2_O_2_. LDH activity was determined in the medium using the colorimetric method (Cytotoxicity Detection Kit, Roche Diagnostic GmbH, Mannheim, Germany). The LDH released into the medium acts as the marker of cellular toxicity. According to this test, the amount of colored hydrazone formed after the reaction of pyruvic acid with 2,4-dinitrophenylhydrazine is inversely proportional to the activity of LDH in the sample, which can be quantified by measuring the absorbance at 420 nm. The results were expressed as the percentage of control cells incubated in the absence of the examined extracts and H_2_O_2_.

### MTT assay

Cytotoxicity was measured as previously described [22]. The method is based on the reducing capacity of the living cells, which is estimated through the presence of insoluble intracellular formazan crystals. The amount of formazan crystals formed depends on the activity of intracellular dehydrogenases and is independent of changes in the integrity of the plasma membrane [24]. SH-SY5Y cells were incubated with MTT (Sigma-Aldrich) for 3 h at 37°C. MTT was prepared in PBS and added at a final concentration of 0.15 mg/mL. Then, the formazan crystals were dissolved in dimethyl sulfoxide and the absorbance of each sample was measured at 570 nm in a Multiscan plate-reader (Labsystems, Vantaa, Finland). The results were expressed as the percentage of control cells incubated in the absence of the examined extracts and H_2_O_2_.

### Ethics

The animals were handled following the European Union Guidelines on animal care (Directive 2010/63/EU, 2010 European Union, 2010 Directive 2010/63/EU, 2010). The experimental design was approved by the Local Ethics Commission in Lublin (permission number 68/2018).

### Animals and sampling

In this study, six clinically healthy female Holstein–Friesian female calves at the age of 4–8 weeks were used. The experiment was conducted in the vivarium of the National Veterinary Research Institute, Pulawy. The animals were randomly assigned to two equal groups, experimental (E) and control (C), and housed in two pens with free access to water. During the study period, the amount of feed intake, rectal temperature, and overall state of health of the calves were recorded daily. The body weight (b.w.) gains were monitored weekly until the end of the study (day 49). Once a week, blood samples were collected from the *vena jugularis externa*. A maximum of 39 ml of blood was collected from each animal using single needle insertion to minimize the distress caused to the calves. The experimental procedures and animal management protocols were devised according to the detailed unified requirements of the Local Ethics Committee for Animal Experimentation. This procedure was classified as being of moderate severity and not needing anaesthesia and/or analgesia. For the analysis of selenium concentration in serum, whole blood samples were collected in serum separator tubes (Medlab, Raszyn, Poland) and were subsequently centrifuged at 1500 *g* for 10 min to obtain sera, which was stored at −20°C until analysis. For white blood cell counts and the percentage of lymphocyte subpopulations, whole blood samples were collected in 1 mL vacutainers with K2-EDTA as the anticoagulant (Medlab, Raszyn, Poland) and examined within 1 h.

### ADG of calves

Calves were given 312.5 g of milk replacer suspended in 2.5 L of warm water twice a day from the beginning up to the third week of the experiment and 375 g of milk replacer suspended in 3 L of warm water twice a day and 500 g of mixed calf protein feed once a day from the fourth to the seventh weeks of the experiment. Calves from both groups were fed hay and allowed water *ad libitum*. Table 2 shows the nutrient composition of the diet. In addition, calves from the experimental group were given *L*. *edodes* in the lyophilized form enriched with selenitetriglycerides (50 mg/L of Se(IV)) as a feed additive. The lyophilizate was added to the morning portion of milk replacer at a dosage of 0.005 mg Se(IV)/kg b.w. of calves i.e. 0.086 g of selenium-enriched mushroom lyophilizate/kg b.w. of calves per day for seven weeks. Calves’ b.w. was recorded before the administration of feed additives (0 week of the experiment) and every week from the first to the seventh weeks of the experiment. The ADG was calculated as body weight at the end of the period − body weight at the beginning of the period × period duration^−1^.

**Table 2:** Ingredient and nutrient composition of the calves basal diet.

Ingredient	Value
**Milk replacer**	
Crude protein (%)	20.0
Crude oils and fats (%)	8.0
Ash (%)	6.0
Crude fiber (%)	1.3
Calcium (%)	0.7
Phosphorus (%)	0.45
Sodium (%)	0.1
Lysine (%)	1.4
Vitamin A (IU/kg)	10,000
Vitamin D_3_ (IU/kg)	2000
Vitamin E (mg/kg)	80
Vitamin K_3_ (mg/kg)	1.0
Vitamin B1 (mg/kg)	4.3
Vitamin B2 (mg/kg)	4.3
Vitamin B6 (mg/kg)	4.3
Vitamin C (mg/kg)	100
Niacinamide (mg/kg)	6.6
Calcium D-pantothenate (mg/kg)	8.6
Folic acid (mg/kg)	0.35
Vitamin B12 (mg/kg)	0.05
Biotin (mg/kg)	0.07
Choline chloride (mg/kg)	300
Manganese (mg/kg)	64
Zinc (mg/kg)	56
Iron (mg/kg)	80
Copper (mg/kg)	8
Iodine (mg/kg)	0.96
Selenium (mg/kg)	0.2
*Enterococcus faecium* (cfu/kg)	1.2 × 10^9^
**Mixed calf feed**	
Crude protein (%)	18.5
Crude oils and fats (%)	3.3
Crude fiber max. (%)	6.5
Crude ash max. (%)	9.0
Phosphorus (%)	0.8
Calcium (%)	1.3
Sodium (%)	0.23
Magnesium (%)	0.25
Vitamin A (IU/kg)	25,000
Vitamin D_3_ (IU/kg)	5000
Vitamin E (mg/kg)	25.0

cfu–colony-forming unit.

### Serum selenium concentration

Selenium concentration in serum samples was analyzed by means of inductively coupled plasma mass spectrometry (ICP-MS) with the use of Varian 820 MS, Bruker M 90 and Plasma Quant ICP-MS (all Analytik Jena, Jena, Germany) and Varian Vista Pro (Agilent, Santa Clara, CA, USA) and iCAP Duo 7000 (ThermoFisher, Waltham, MA, USA) ICP optical emissions spectrometers.

### Blood leukocyte counts

Total WBC (white blood cell) counts with LYM (lymphocyte), MON (monocyte), and GRA (granulocyte) leukocyte differentiation were examined in peripheral blood using an Exigo automatic veterinary blood analyzer (Boule Medical AB, Spånga, Sweden).

### CDs and WC4 antigens

The percentage of lymphocyte subpopulations containing surface antigens CD2^+^ (T cells), CD4^+^ (T helper cells), CD8^+^ (T suppressor/cytotoxic cells), and WC4^+^ (B cells) was analyzed with an EPICS XL 4C flow cytometer (Beckman Coulter Company, Brea, CA, USA). The expression of CD markers was determined using the following primary mouse anti-bovine monoclonal antibodies (mAb): anti-bovine CD2 fluorescein isothiocyanate isomer 1 (FITC), anti-bovine CD4 FITC, and anti-bovine CD8 FITC. The expression of WC4^+^ was determined using the following antibodies: anti-bovine WC4 and rabbit F(ab’)2 anti-mouse IgG secondary polyclonal antibody. To standardize the analysis, anti-bovine CD45 FITC and cross-reacting anti-human CD14 R- Phycoerythrin-Cy5 (RPE-Cy5) mouse mAb were used (Bio-Rad, Hercules, CA, USA). Whole blood samples (100 μL) were incubated at room temperature (18–25°C) with appropriate mAb for 15 min, and erythrocytes were lysed using lysis fluid for 20 min. For the analysis of WC4^+^, the samples were incubated for 30 min with the appropriate primary mAb, washed with PBS, and incubated for 15 min with the secondary antibody. After washing the samples with PBS containing 5% inactivated fetal calf serum, the mixture was suspended in 500 μL of the same PBS and analyzed with the use of the EPICS XL 4C flow cytometer. The data were obtained as listmodes in histogram form with the use of SYSTEM II 3.0 software (Beckman Coulter).

### Statistical analysis

Tukey’s test was applied to the results of selenium content. P<0.05 was assumed for all tests, which were carried out in InStat software (GraphPad Software, San Diego, CA, USA). The cytotoxicity results in MTT and LDH assays were expressed as mean ± SEM (standard error of mean). The significance of differences between the means was evaluated using the Bonferroni post-hoc test followed by analysis of variance. The results of the experiment on animals are presented as arithmetic mean ± standard deviation (SD). The differences between the mean values recorded in the experimental and control groups were analyzed using the *t*-test with a statistically significant level of P<0.05.

## Results

### Optimization of liquid culture of *L*. *edodes* mycelium

According to our results, effective growth of the biomass of *L*. *edodes* was obtained from aerated liquid cultures on the Oddoux medium both with and without the addition of selenitetriglycerides at 25 ± 2°C under the natural photoperiod and within the experimental 10-day growth cycle. Aeration and the removal of carbon dioxide were found to be effective in the optimization of culture conditions. These facilitators resulted in maximal growth of the mycelium, which was obtained within 10 days of culture and markedly sooner than was seen for the control cultures without aeration (which took about 21 days to grow).

There was a 30% increase in the growth of *L*. *edodes* biomass in selenitetriglyceride-enriched culture over growth in unenriched culture. The yield of the biomass grown on the unenriched modified Oddoux medium averaged about 10.6 g d.w./L, but with the addition of selenitetriglyceride and the advantage of aerated conditions, it was about 15.1 g d.w./L [25].

### Selenium accumulation *in L*. *edodes* mycelium enriched with selenitetriglycerides

Selenium accumulation was tested in the mycelium of *L*. *edodes* grown on the medium with the addition of selenitetriglycerides. In the commercially obtained *L*. *edodes* fruiting bodies, which in turn were obtained from the natural habitat, the selenium content was found to be similar to the content in the controls, which amounted to 0.01 mg/100 g d.w. Higher concentrations of selenium were obtained in the mycelium of *L*. *edodes* from liquid cultures, which contained 0.79 mg/100 g d.w. (Table 3).

**Table 3:** Selenium content in *L*. *edodes* with and without the addition of selenitetriglycerides.

*L*. *edodes* part and culture media (Samples)	Se (mg/100g DW)
Basal medium (unenriched)	—
Fruiting bodies (unenriched)	0.01±0.00 ^a^
Mycelium (unenriched and control)	0.79±0.63 ^a^^,^^b^
Mycelium + Se(IV) 25 mg/L medium	192.65±5.12 ^a^^,^^b^
Mycelium + Se(IV) 50 mg/L medium	532.31±35.53 ^a^^,^^b^^,^^c^^,^^d^
Medium + Se(IV) 25 mg/L medium	95.68±2.18 ^a^^,^^b^^,^^c^^,^^d^^,^^e^
Medium + Se(IV) 50 mg/L medium	183.97±8.70 ^a^^,^^b^^,^^c^^,^^d^^,^^e^

Mean ± standard deviation; n = 6;—–not detected; Tukey’s test was used to reveal the differences between paired groups of selenium in rows

a,b,c,d,e–values with different letters differ significantly (P<0.05)

In *L*. *edodes* mycelium supplemented with selenium, considerable accumulation of this element (from 192.6 to 532.3 mg/100 g d.w.) was detected (Table 3). This shows that the higher the concentration of selenium in the medium the higher the increase in the amount of 332 selenium in the mycelium of *L*. *edodes*.

### Cytotoxicity test

**Fig 1:**
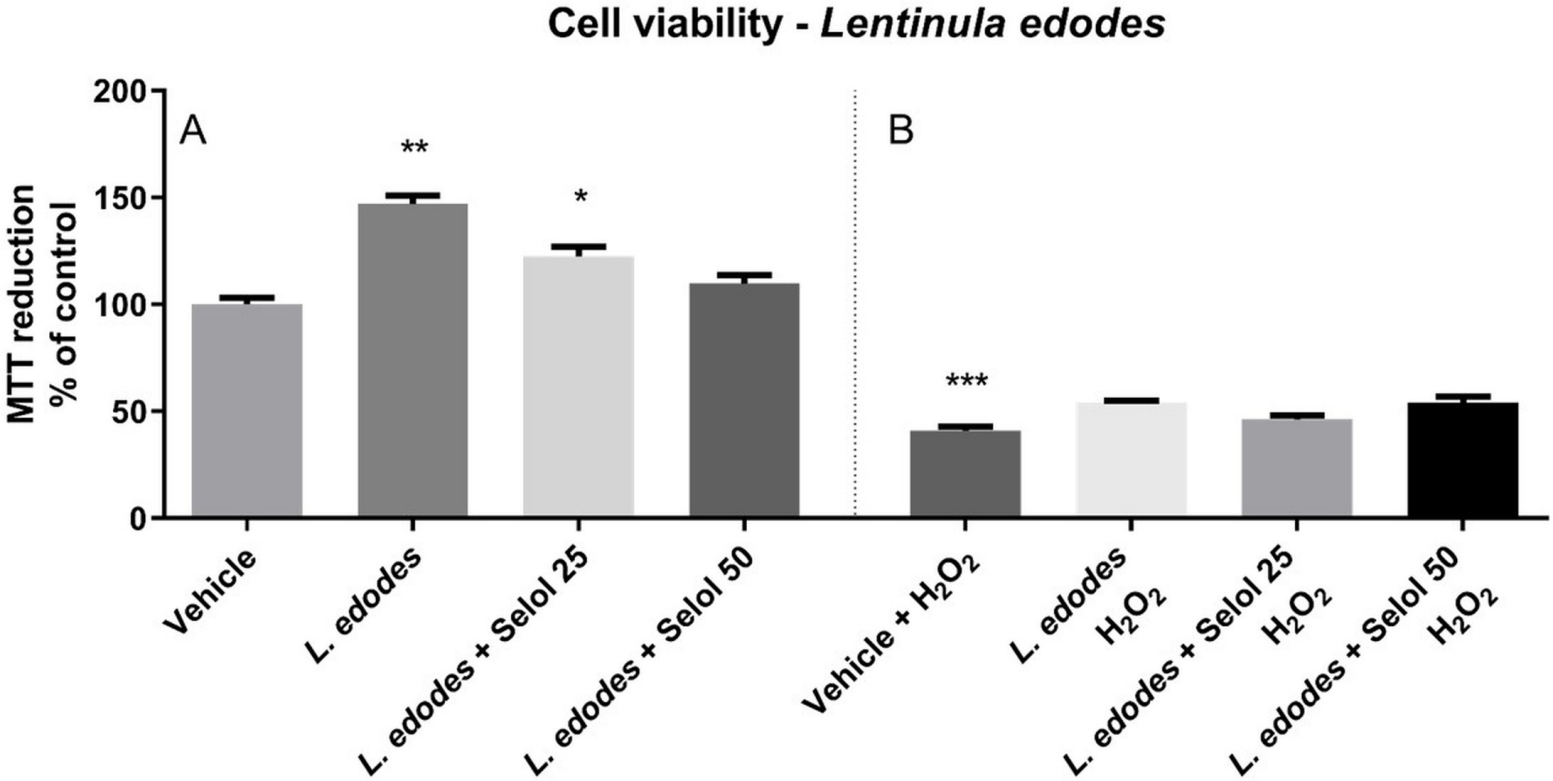
The effect of *L*. *edodes* extract with or without selenitetriglyceride supplementation on (A) the basal and (B) H_2_O_2_-induced 3-(4,5-dimethylthiazol-2-yl)-2,5-diphenyltetrazolium bromide (MTT) reduction in SH-SY5Y human neuroblastoma cell line. Results are shown as the percentage of control cells not exposed to the extract of *L*. *edodes* and H_2_O_2_. The results are expressed as mean ± standard error of mean. The significance of differences between the means was evaluated using the Bonferroni post-hoc test followed by a one-way analysis of variance (selenitetriglicerides 25 –addition of 25 mg of Se(IV)/L of medium; selenitetriglicerides 50 –addition of 50 mg of Se(IV)/L of medium); *P<0.05; **P<0.01; ***P<0.001 versus control cells exposed to vehicle (1:10 ethanol/H_2_O); n = 12).

**Fig 2:**
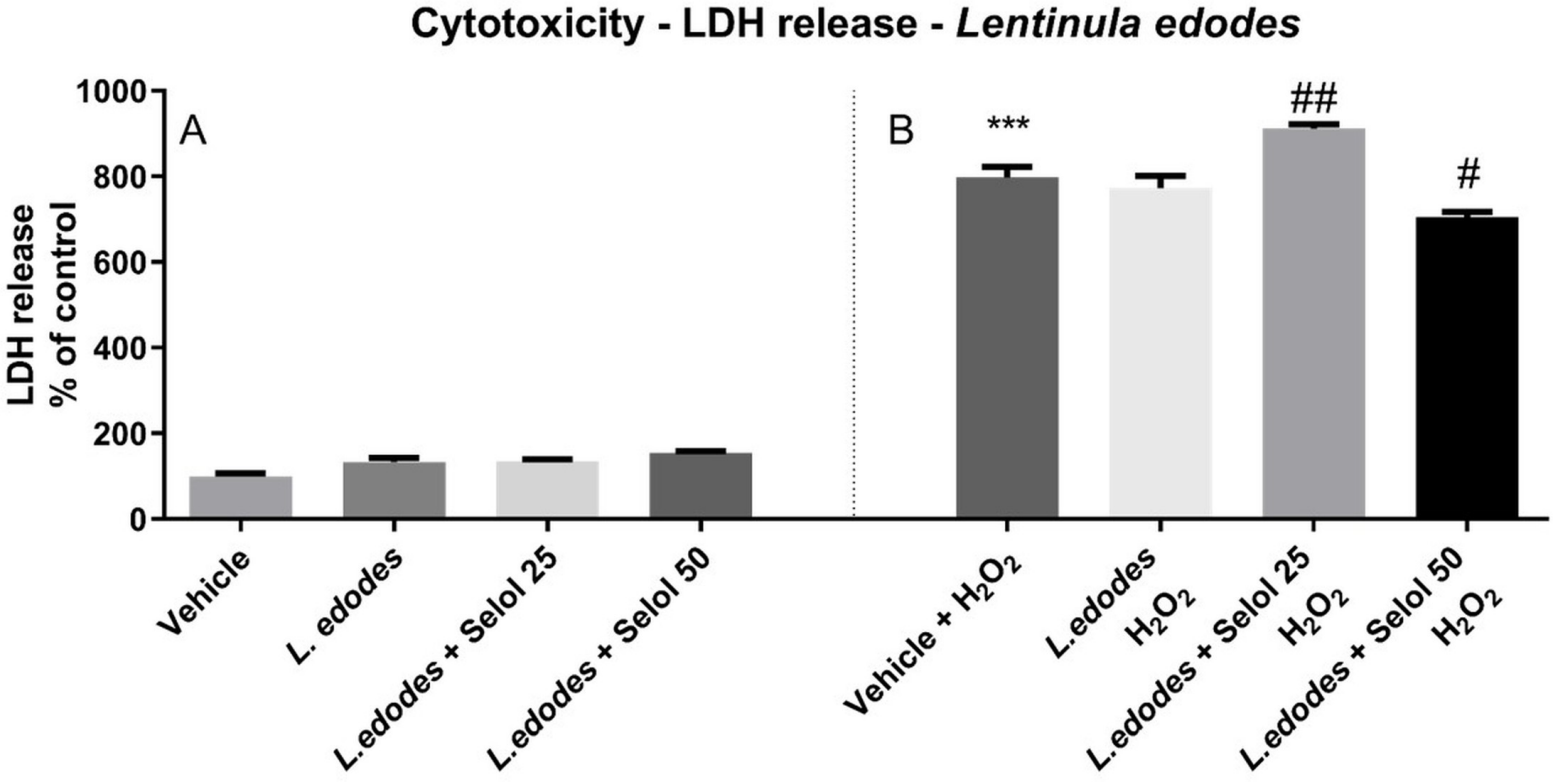
The effect of *L*. *edodes* extract with and without selenitetriglyceride supplementation on (A) the basal and (B) H_2_O_2_-induced release of lactate dehydrogenase (LDH) in SH-SY5Y human neuroblastoma cell line. Results are shown as the percentage of control cells not exposed to the extract of *L*. *edodes* and H_2_O_2_. The results are expressed as mean ± standard error of mean. The significance of differences between the means was evaluated using the Bonferroni post-hoc test followed by a one-way analysis of variance (selenitetriglicerides 25 –addition of 25 mg of Se(IV)/L of medium; selenitetriglicerides 50 –addition of 50 mg of Se(IV)/L of medium); ***P<0.001 versus control cells exposed to vehicle (1:10 ethanol/H_2_O); ^#^P<0.05; ^##^P<0.005 versus cells exposed to vehicle + H_2_O_2_ alone; n = 12).

SH-SY5Y cells treated with the extracts of *L*. *edodes* mycelium presented significantly more formazan crystals (i.e., these cells had increased viability). Se(IV) at a concentration of 25 mg/L in the medium also showed similar activity (P<0.01 and P<0.05, respectively). However, *L*. *edodes* cultivated with selenium at a concentration of 50 mg/L of medium did not show any cytoprotective effect (Fig 1). In the LDH release test, SH-SY5Y cells treated with the extracts of *L*. *edodes* mycelium did not release LDH into the medium, regardless of the method of cultivation. However, in the case of cells treated with H_2_O_2_, only extracts of *L*. *edodes* cultured with the addition of Se(IV) at a concentration of 50 mg/L in the medium decreased the amount of cytotoxicity (P<0.05). These studies showed that the tested extracts did not show cytotoxic effect on SH-SY5Y cells, and the extracts of *L*. *edodes* mycelium alone and with an addition of Se (25 mg/L) even increased cell viability (Fig 2).

Our results evinced efficient *L*. *edodes* selenium accumulation. According to the results of cytotoxicity tests, the amount of selenium present in the mycelium was found to be safe and could be used for further experiments.

### Experiment on animals

#### ADG of calves

The calves from the experimental group took all the portions of the examined feed additive (*L*. *edodes* enriched with selenium). The ADG for the whole period of the experiment was 20.41±167.05 g/d in the control group and 193.88 ±107.51 g/d in the experimental group, and these differences were not statistically significant (P<0.05).

#### Serum selenium concentration and immunological parameters of calves

Table 4 shows the mean concentrations of selenium in the serum of calves. In serum samples of the control and experimental animals, these were below reference values according to Idexx Laboratories analytical method ICP-MS (7166) [26] (<80 μg/L) before the beginning of the study. The serum concentration of selenium was significantly higher in calves fed with selenium-enriched mycelium of *L*. *edodes* (P<0.05). A significant increase in the concentration of selenium (from 23 μg/L to 177.67 μg/L) in experimental calves was detected after the first week of supplementation with the feed additive, and the resulting selenium level is considered adequate, falling in the 80–300 μg/L range proposed by Puls [27] (Table 4).

**Table 4:** Effects of dietary treatments on selenium concentrations in blood serum of calves.

Weeks of experiment	Serum concentration of selenium in control group (μg/L)	Serum concentration of selenium in experimental group (μg/L)
0	19.33±1.67	23.00±9.50
I	23.00±3.51 ^a^	177.67±24.39 ^b^
II	23.33±4.18 ^a^	175.00±18.15 ^b^
III	27.00±3.51 ^a^	236.00±19.86 ^b^
IV	23.67±2.40 ^a^	177.67±19.17 ^b^
V	33.67±7.06 ^a^	138.33±5.17 ^b^
VI	33.00±3.46 ^a^	178.67±6.89 ^b^
VII	38.50 ±5.48 ^a^	210.67 ±2.90 ^b^

Mean ± standard deviation; n = 3

^a,b^–values with different letters differ significantly (P<0.05).

#### Blood leukocyte counts

**Fig 3:**
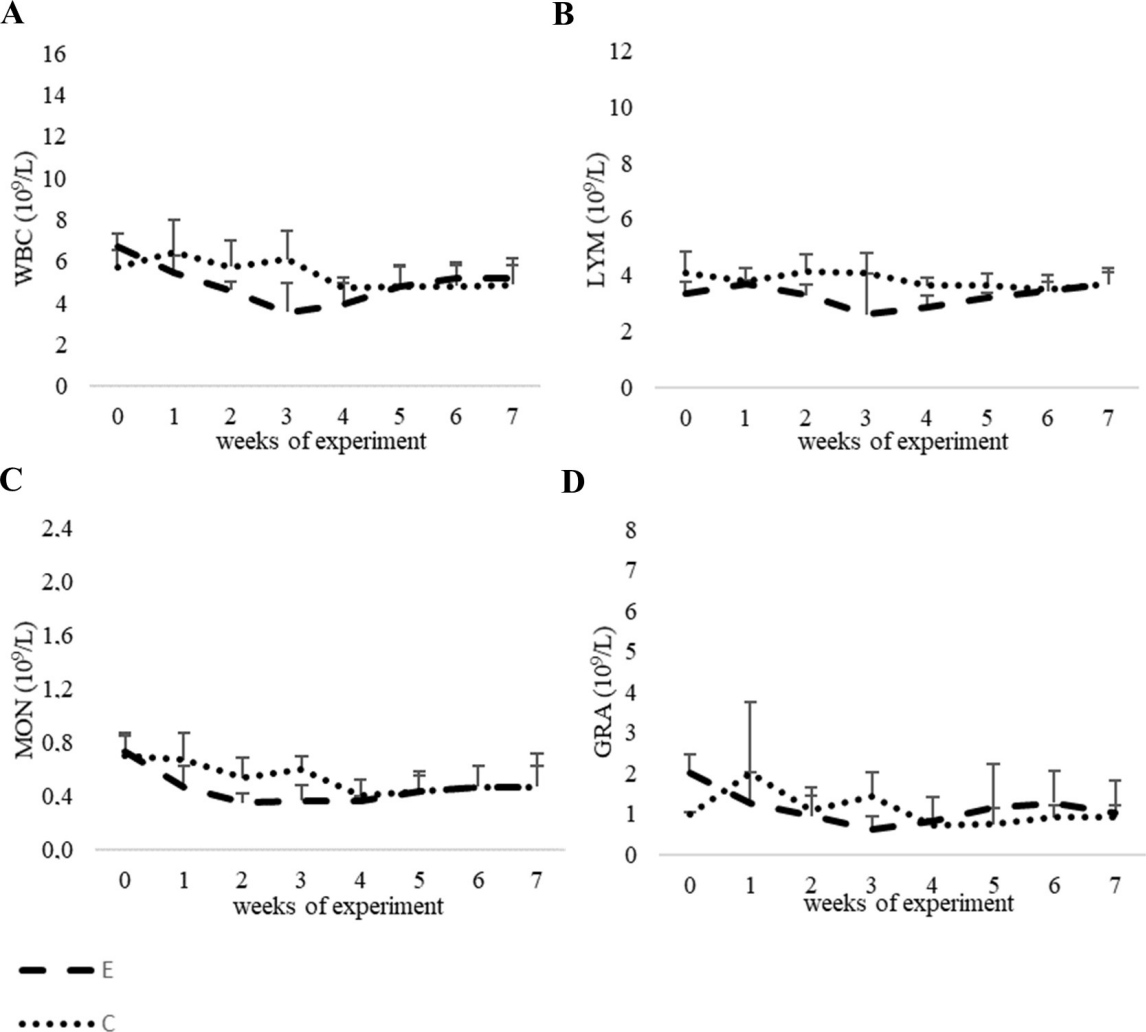
3A The results of a total count of white blood cells (WBC); 3B with leukocyte differentiation (LYM); 3C monocyte differentiation (MON); 3D and granulocyte differentiation (GRA) in the peripheral blood of calves ± SD (10^9^ cells/L).

Our results showed a slight decrease in white blood cell counts and their subsets (i.e. LYM and MON) in the experimental group between the second and third weeks of the experiment, whereas from the fourth and fifth weeks the counts had similar values to those of the control group (Fig 3, S1 Table). The GRA values were comparable in both groups of animals throughout the study. No statistically significant differences were found between the experimental and control groups and the results were within the reference range.

#### CDs and WC4 antigens

**Fig 4:**
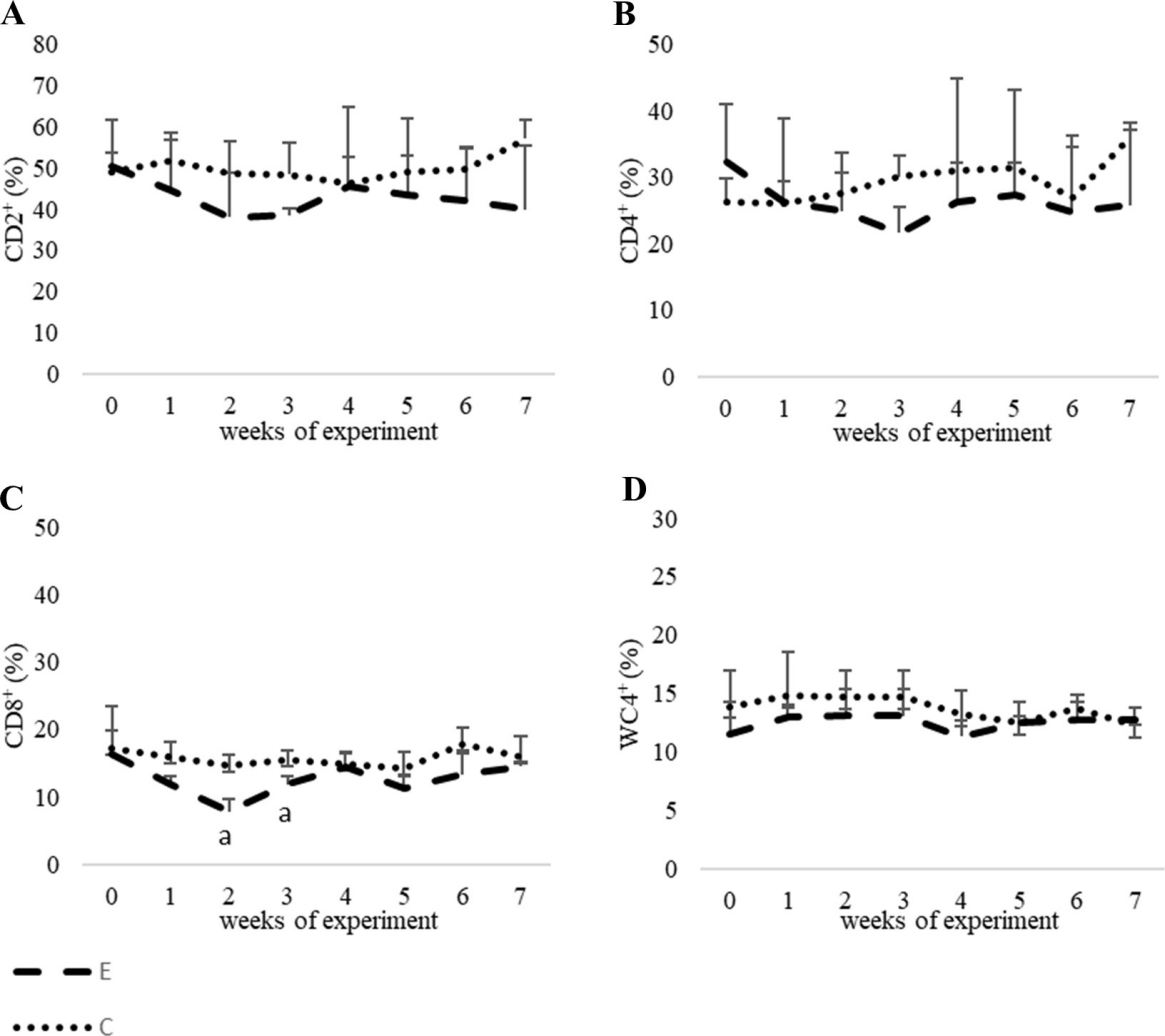
The results of examination of the percentage of lymphocyte subpopulations in peripheral blood of calves (%) ± SD. 4A CD2^+^; 4B CD4^+^; 4C CD8^+^; 4D WC4^+^ ± SD; E–experimental calves; C–control calves. ^a^–a value with this letter differs significantly (P<0.05).

In the experimental group, the percentage of CD2^+^ and CD4^+^ was slightly lower than that of the control group between the first and third and between the second and fifth weeks of the study respectively. In both cases no statistically significant differences were found. The percentage of CD8^+^ cells in the experimental group was generally lower throughout study with the exception of the fourth and seventh weeks. Statistically significant differences were observed in the second and third weeks when compared to the control group (P<0.05). The percentage of WC4^+^ was slightly lower up to the fourth week of the study in the experimental group than in the control group. In the remaining weeks of the study, the percentage was similar to that of the control group. All the values were within the reference range and no statistically significant differences were observed (Fig 4,S1 Table).

## Discussion

Selenium is known to modify and regulate redox processes depending on the needs of an organism. The selenium in the selenium-enriched *L*. *edodes* mycelium exhibited a therapeutic effect; therefore, we can say that it is safe for use, despite its low therapeutic index [28, 29]. Selenium in the form of selenitetriglycerides taken up by *L*. *edodes* and accumulated in the mycelium constitutes a good source of selenium for the production of livestock feed and animal protein for human consumption. Its incorporation in mushrooms may enhance their immunostimulatory, antioxidative, and anti-inflammatory effects [18, 30, 31]. In this study, selenium in the mycelium of *L*. *edodes* ranged from 95.7 to 532.3 mg/100 g d.w. depending on its concentration in the medium. For comparison, vegetable foods contain selenium from 0.02 to 0.12 μg/g d.w., whereas marine fishes and seafood contain higher amounts than vegetable foods (0.56 to 2.00 μg/g d.w.) [1].

The safety of mycelium enriched with Se(IV) in the form of the organic complex—selenitetriglycerides—was tested based on the evaluation of cytotoxicity using MTT and LDH tests. These tests are routinely used to assess the cytotoxicity of medicinal mushrooms [23]. The investigations showed that the tested extracts did not exert a cytotoxic effect on SH-SY5Y cells, and that the extracts of *L*. *edodes* mycelium alone and with the addition of Se (25 mg/L) even increased cell viability. LDH and MTT tests showed that the three extracts of *L*. *edodes* tested had no cytotoxic activity on SH-SY5Y cells, and the latter test determined that the extracts of *L*. *edodes* mycelium alone and with the addition of Se (25 mg/L) even increased cell viability. For cells damaged by the addition of H_2_O_2_ (an *in vitro* oxidative stress model) the tested extracts did not intensify the H_2_O_2_-induced loss in SH-SY5Y cell viability; *L*. *edodes* mycelium alone had no effect on H_2_O_2_-induced cell membrane damage, as determined by the LDH release, the mycelium with the addition of Se (50 mg/L) had a protective effect, while the extract of *L*. *edodes* with addition of Se (25 mg/L) enhanced the cytotoxic action of H_2_O_2_.

Based on the literature, selenium deficiency is observed in 23% of dairy cows and 31% of calves in the Czech Republic [31]. Our experiment confirmed that similar problem may affect calves in Poland as the study animals both from control and experimental groups on the first day of the trial had serum selenium concentrations below the reference value. These results indicate that enrichment of mycelium of *L*. *edodes* was useful in significantly increasing the level of selenium in the serum of calves. There are studies confirming that a long-term administration of organic selenium resulted in higher Se blood concentration than a few repeated parenteral administrations of inorganic selenium [32]. Pehrson et al. [33], compared two selenium supplements in calves: with inorganic selenium (as selenite) and with the form of organic selenium from yeast (as selenomethionine). Both groups had free access to the supplement, which contained 30 mg of Se/kg. In the selenomethionine group, 10 out of 11 calves had serum selenium concentrations of <100 μg/L. In the selenite group, seven out of nine calves had <100 μg/L and two out of nine calves had <50 μg/L serum selenium concentrations. The amount of selenium in whole blood of the cows and calves in the selenomethionine group was higher than that in the animals from the selenite group, which indicates that organic selenium is absorbed better than inorganic. These findings agree with those reported by Ran, who posited that supplementation of the diet with selenium-enriched yeast appeared to be more effective than supplementation with sodium selenite [34]. Similar results were obtained by Gunter et al. [32] and Rossi et al. [35] who noted that supplementation with selenium-enriched yeast compared with sodium selenite at a concentration of 0.32 mg/kg d.w. improved serum selenium concentration significantly (118.62 μg/L vs. 108.64). These results also confirm that yeast selenium is better absorbed than sodium selenite.

Supplementing the feed for cattle with selenium is important for improving calves’ health and production efficiency and is also important to enrich animal products intended for human consumption. Currently, scientific research is being conducted on the precise dose and form of selenium that can be fed to calves. The effects of selenomethionine and sodium selenate are well understood; however, alternative forms are still being sought. Studies are being conducted on encapsulation to protect selenium from adverse effects of the rumen environment and on the use of nanoparticles or fungi or algae grown in a selenium-enriched environment for incorporation into cellular structures [36, 37, 38]. Studies conducted by Grilli et al. [36] showed that lipid microencapsulation can protect selenium from rumen utilization and that this form is incorporated into milk more efficiently than that of the free form. His study also indicated that selenium supplementation with the highest doses (5 mg/kg fed) in microencapsulated form significantly increased the level of selenium in the blood compared to supplementation with selenium-enriched yeast. Selenium injection is also considered a very useful method to improve the level of selenium in deficient calves. However, it should not be used as a permanent source of selenium because of the risk of anaphylactic reaction [39]. Most cases recover but fatal effects affect 10–20% of calves [40]. Furthermore, selenium injections are not allowed for continuous use in the increasingly popular organic livestock production [41]. Chorfi [39] measured the effect of selenium injections while adding sodium selenite to both the experimental and control animals’ diets to provide 3 mg Se/animal/day only in ingested form. Heifers which received selenium injections showed significantly higher concentrations of plasma and whole blood selenium and glutathione peroxidase activity than those of control group animals. In another study, cows administered a mixture of 30 mg selenium in the form of sodium selenite and calves injected with 5.5 mg selenium and 75 IU E/100 kg also had significantly higher plasma concentrations of selenium and whole blood glutathione peroxidase activity than control animals had [42]. Research results indicate that the additive used is a good and safe source of selenium for cattle.

The tendency towards larger b.w. gains in the experimental group of calves than in the control group in this study is in accordance with the results obtained by Ebrahimi et al. [43]. They showed superior b.w. gains of Holstein male calves that were fed with organic selenium (0.3 mg Sel-Plex per kg dry matter added to milk of suckling calves), but similarly to this study, could not show statistically significant differences. However, it is difficult to compare these test results because the selenium content was given per kg dry matter. Another study was conducted by Wichtel et. al. [44] on Friesian heifers that were fed with pasture and received two intraruminal selenium pellets which released Se at the rate of about 3 mg/d. The authors obtained 20% higher body weight gains in calves supplemented with selenium when compared to the control group. There is also study that describes the results of single or multiple subcutaneous injections of inorganic Se in newborn calves. In that experiment, calves that received multiple injections of Se had significantly greater ADG than control calves, but in calves given only a single injection it was not significantly different from that of the controls [45]. Similarly, a single intramuscular injection of selenium also had no effect on calves’ growth performance, although this kind of administration efficiently prevented deaths from muscular dystrophy [46]. It is worth noting that in the aforementioned papers, animals were supplemented with other forms of selenium than those used in this study. To the best of our knowledge, there are no other data concerning the effect of consumption of *L*. *edodes* enriched with selenium on body weight gains in calves.

According to a previous study, in humans, selenium can be accumulated in lymphocytes, macrophages and neutrophils and subsequently it can improve humoral and cell-mediated immune response. Usually, small doses of selenium stimulate an immune response, and excessive doses suppress it [4, 47 48]. Our WBC analysis with lymphocyte immunophenotyping agrees with that of those who stated that there was no significant effect of selenium on hematological parameters and that possible differences in hematological parameters need only be considered to have limited biological meaning [49]. In our study no significant effect on WBC counts was observed. However, there are studies which suggest that although selenium is considered beneficial for the immune system, leukocyte function is often not affected by its supplementation [3, 48]. In the study of Machado et al. [50] it was shown that in Holstein cows that were subcutaneously supplemented with inorganic Se, the leukocyte function was not affected. Similarly, in the study of Weiss et al. [51], although the mean concentrations of Se in serum samples of cows and newborn calves were higher in the group fed with organic selenium (Se-yeast) than with an inorganic form (selenite), differences in neutrophil function were not observed between the groups.

Regarding *L*. *edodes* mycelium, there are numerous studies which describe improved immunity in humans as well as animals when mycelium was regularly consumed. Dai et al. [52] describes that consuming *L*. *edodes* mushrooms improves human immunity and it was seen by increased T-cell proliferation and activation. More studies concern the immune function of white blood cells potentiated by mycelium extracts. For example Lee et al. [53] examined innate immunity in poultry and found it stimulated after consumption of an extract of *L*. *edodes*. Another study shows that polysaccharide isolated from *L*. *edodes* stimulates macrophage activity [54]. Therefore, it is difficult to compare existing results univocally with our own results. To our knowledge there are no data about lymphocyte immunophenotyping of calves fed with *L*. *edodes* mycelium enriched with selenium. Additionally, Yu et al. [55] claims that that it is not surprising that the effect of consumption of edible mushrooms on induced inflammatory responses is only modest because commonly consumed food should not exert a strong influence on the immune system, moreover it could be harmful to have a staple dietary component which induces or suppresses immune function.

In this study, a beneficial effect of *L*. *edodes* enriched with selenium on calves was observed in an upward tendency in the b.w. gains and in an increase of selenium concentration in sera.

## Conclusion

Selenium-enriched mycelium of *L*. *edodes* is characterized by the absence of any cytotoxic effect, which is advantageous for its dietary administration to control animal selenium deficiencies. In this study, the consumption of the *L*. *edodes* mycelium resulted in an increase in the b.w. and the establishment of an adequate level of selenium in the serum of calves that had deficiencies at the beginning of the study. The WBC counts and percentages of the lymphocyte subpopulations in the experimental calves lying within the physiological range confirmed a lack of adverse effect of the *L*. *edodes* mycelium on the non-specific immune response in the examined animals. Further study is required to evaluate the effect of *L*. *edodes* enriched with selenium on other parameters of immune response in calves.

## Supporting information

S1 Table (DOCX)

## References

[1] Muszyńska B, Kała K, Włodarczyk A, Krakowska A, Ostachowicz B, Gdula-Argasińska J, et al *Lentinula edodes* as a source of bioelements released into artificial digestive juices and potential anti-inflammatory material. Biol Trace Elem Res. 2020;194(2): 603–613. 10.1007/s12011-019-01782-8 31256391 PMC7015957

[2] Hefnawy AEG, Tórtora-Pérez JL. The importance of selenium and the effects of its deficiency in animal health. Small Rumin Res. 2010;89(2–3): 185–192. 10.1016/j.smallrumres.2009.12.042

[3] Sordillo LM. Selenium-dependent regulation of oxidative stress and immunity in periparturient dairy cattle. Vet Med Int. 2013;Article ID 154045. 5/2013/1155.1. 10.1155/2013/154045 PMC3557619 23401850

[4] Kiremidjian-Schumacher L, Stotzky G. Selenium and immune responses. Environ Res. 1987;42(2): 277–303. 10.1016/s0013-9351(87)80194-9 3552651

[5] Mehdi Y, Dufrasne I. Selenium in Cattle: A Review. Molecules. 2016;21(4): 545 10.3390/molecules21040545 27120589 PMC6274551

[6] Panev A, Hauptmanová K, Pavlata L, Pechová A, Filípek J, Dvořák R. Effect of supplementation of various selenium forms and doses on selected parameters of ruminal fluid and blood in sheep. Czech J Anim Sci. 2013;58(1):37–46. 10.17221/6524-cjas

[7] Pechova A, Pavlata L, Illek J. Blood and tissue selenium determination by hydride generation atomic absorption spectrophotometry. Acta Vet Brno. 2005;74: 483–490. 10.2754/avb200574040483

[8] Kała K, Muszyńska B, Zając M, Krężałek R, Opoka W. Determination of zinc(II) ions released into artificial digestive juices from culinary-medicinal button mushroom, *Agaricus bisporus* (Agaricomycetidae), biomass of in vitro cultures using an anodic stripping voltammetry method. Int J Med Mushrooms. 2016;18(2): 155–164. 10.1615/IntJMedMushrooms.v18.i2.60 27279537

[9] Grzywacz A, Gdula-Argasińska J, Kała K, Opoka W, Muszyńska B. Anti-inflamatory activity of biomass extracts of the Bay mushroom *Imleria badia* (Agaricomycetes), in RAW 264.7 cells. Int J Med Mushrooms. 2016;18(9): 769–779. 10.1615/IntJMedMushrooms.v18.i9.20 27910769

[10] Muszyńska B, Kała K, Sułkowska-Ziaja K. Edible mushrooms and their in vitro culture as a source of anticancer compounds In: Malik S, editor. Biotechnology and production of anti-cancer compounds. Brazil: Springer; 2017 p. 231–251.

[11] Mao G, Feng W, Xiao H, Zhao T, Li F, Zou Y, et al Purification, characterization, and antioxidant activities of selenium-containing proteins and polysaccharides in royal sun mushroom, *Agaricus brasiliensis* (Higher Basidiomycetes). Int J Med Mushrooms. 2014;164: 63–75. 10.1615/intjmedmushrooms.v16.i5.50 25271981

[12] Bederska-Łojewska D, Świątkiewicz S, Muszyńska B. The use of Basidiomycota mushrooms in poultry nutrition–A review. Anim Feed Sci Technol. 2017;230: 59–69. 10.1016/j.anifeedsci.2017.06.001

[13] Akramiene D, Kondrotas A, Didziapetriene J, Kevelaitis E. Effects of beta-glucans on the immune system. Medicina (Kaunas). 2007;43(8): 597–606. 17895634

[14] Muta T. Molecular basis for invertebrate innate immune recognition of (1—>3)-β-D-glucan as a pathogen-associated molecular pattern. Curr Pharm Des. 2006;12(32): 4155–4161. 10.2174/138161206778743529 17100618

[15] Rincão VP, Yamamoto KA, Ricardo NMPS, Soares SA, Meirelles LDP, Nozawa C, et al Polysaccharide and extracts from Lentinula edodes: structural features and antiviral activity. Virol J. 2012;9(37). 10.1186/1743-422X-9-37 22336004 PMC3292946

[16] Drori A, Shabat Y, Ben Ya’acov A, Danay O, Levanon D, Zolotarov L, et al Extracts from *Lentinula edodes* (Shiitake) edible mushrooms enriched with vitamin D exert an anti-inflammatory hepatoprotective effect. J Med Food. 2016;19(4): 383–389. 10.1089/jmf.2015.0111 27027234

[17] Morales D, Gil-Ramirez A, Smiderle FR, Piris AJ, Ruiz-Rodriguez A, Soler-Rivas C. Vitamin D-enriched extracts obtained from shiitake mushrooms (Lentinula edodes) by supercritical fluid extraction and UV-irradiation. Innov Food Sci Emerg Technol. 2017;41: 330–333. 10.1016/j.ifset.2017.04.008

[18] Turlo J, Gutkowska B, Herold F, Dawidowski M, Słowiński T, Zobel A. Relationship between selenium accumulation and mycelial cell composition in Lentinula edodes (Berk.) cultures. J Toxicol Environ Health Part A. 2010;73(17–18): 1211–1219. 10.1080/15287394.2010.492005 20706946

[19] Fitak B, Grabowski M, Suchocki P, inventors; Preparat przeciwnowotworowy i sposób jego wytwarzania. Polish patent 176530 (Cl. A61K31/095).1999 June 30.

[20] Suchocki P, Misiewicz-Krzemińska I, Skupińska K, Niedźwiecka K, Lubelska K, Fijałek Z, et al Selenitetriglicerydes affect CYP1A1 and QR activity by involvement of reactive oxygen species and Nrf2 transcription factor. Pharmacol Rep. 2010;62(2): 352–361. 10.1016/s1734-1140(10)70275-9 20508291

[21] Oddoux L. Recherches sur les mycéliums secondaires des Homobasidiésen culture pure. Imprimerie de Trevoux Lyon, France; 1957.

[22] Łojewski M, Pomierny B, Muszyńska B, Krzyżanowska W, Budziszewska B, Szewczyk A. Protective effects of *Bacopa monnieri* on hydrogen peroxide and staurosporine: induced damage of human neuroblastoma SH-SY5Y cells. Planta Med. 2016;82(3): 205–210. 10.1055/s-0035-1558166 26544120

[23] Kim S, Jakhar R, Kang SC. Apoptotic properties of polysaccharide isolated from fruiting bodies of medicinal mushroom *Fomes fomentarius* in human lung carcinoma cell line. Saudi J Biol Sci. 2015;22(4): 484–490. 10.1016/j.sjbs.2014.11.022 26150756 PMC4487262

[24] Regulska M, Pomierny B, Basta-Kaim A, Starek A, Filip M, Lasoń W, et al Effects of ethylene glycol ethers on cell viability in the human neuroblastoma SH-SY5Y cell line. Pharmacol Rep. 2010;62: 1243–1249. 10.1016/s1734-1140(10)70389-3 21273685

[25] Muszyńska B, Kała K, Włodarczyk A, Krakowska A, Ostachowicz B, Gdula-Argasinska J, et al *Lentinula edodes* as a source of bioelements released into artificial digestive juices and potential anti-inflammatory material. Biol Trace Elem Res. 2020;194(2): 603–613. 10.1007/s12011-019-01782-8 31256391 PMC7015957

[26] Idexx Laboratories. Analytical method: ICP-MS (7166). Available from: https://www.idexx.com/en/veterinary/reference-laboratories/tests-and-services/

[27] Puls R. Mineral levels in animal health Diagnostic data. Trinity Western University Press Sherpa International, Clearbrook, Canada; 1988.

[28] Muszyńska B, Grzywacz A, Kała K, Gdula-Argasińska J. The anti-inflammatory potential of *in vitro* cultures of white button mushroom, *Agaricus bisporus* (Agaricomycetes) in CaCo-2 cells. Int J Med Mushrooms. 2018;20(2): 129–139. 10.1615/IntJMedMushrooms.2018025408 29773005

[29] Flis A, Suchocki P, Królikowska M, Suchocka Z, Remiszewska M, Śliwka L, et al Selenitetriglycerides-Redox-active agents. Pharmacol Rep. 2015;67(1): 1–8. 10.1016/j.pharep.2014.07.017 25560568

[30] Muszyńska B, Bederska D, Zięba P. The importance of selenium in the human diet—in the aspect of feeding farm animals. Roczniki Naukowe Zootechniki. 2018;45(2): 135–142.

[31] Książek I, Sitarz K, Rosłon M, Anuszewska E, Hoser G, Dudkiewicz-Wilczyńska J, et al The influence of an organic selenium(IV) compound on progression of tumor induced using prostate cancer cells and gene expression connected to the oxidative stress response. World J Pharm Sci. 2015;2(10): 1146–1158.

[32] Gunter SA, Beck PA, Hallford DM. Effects of supplementary selenium source on the blood parameters in beef cows and their nursing calves. Biol Trace Elem Res. 2013;152(2): 204–211. 10.1007/s12011-013-9620-0 23381681

[33] Pehrson B, Ortman K, Madjid N, Trafikowska U. The influence of dietary selenium as selenium yeast or sodium selenite on the concentration of selenium in the milk of suckler cows and on the selenium status of their calves. J Anim Sci. 1999;77: 3371–3376. 10.2527/1999.77123371x 10641886

[34] Ran L, Wu X, Shen X, Zhang K, Ren F, Huang K. Effects of selenium form on blood and milk selenium concentrations, milk component and milk fatty acid composition in dairy cows. J Sci Food Agric. 2010;90(13): 2214–2219. 10.1002/jsfa.4073 20629108

[35] Rossi CS, Compiani R, Baldi G, Muraro M, Marden JP, Rossi R, et al Organic selenium supplementation improves growth parameters, immune and antioxidant status of newly received beef cattle. J Anim Feed Sci. 2017;26(2): 100–108. 10.22358/jafs/70765/2017

[36] Grilli E, Gallo A, Fustini M, Fantinati P, Piva A. Microencapsulated sodium selenite supplemen-tation in dairy cows: Effects on selenium status. Animal. 2013;7(12): 1944–1949. 10.1017/S1751731113001547 24016452

[37] Doucha J, Lívanský K, Kotrbáček V, Zachleder V. Production of *Chlorella* biomass enriched by selenium and its use in animal nutrition: A review. Appl Microbiol Biotech. 2009;83: 1001–1008. 10.1007/s00253-009-2058-9 19533119

[38] Pelyhe C, Mézes M. Myths and facts about the effects of nano selenium in farm animals–mini-review. Euro Chem Bullet. 2013;2: 1049–1052. 10.17628/ecb.2013.2.1049-1052

[39] Chorfi Y, Girard V, Fournier A, Couture Y. Effect of subcutaneous selenium injection and supplementary selenium source on blood selenium and glutathione peroxidase in feedlot heifers. Can Vet J. 2011;52(10): 1089–1094. 22467963 PMC3174504

[40] Divers TJ, Peek SF. Rebhun's Diseases of Dairy Cattle. 2nd ed W.B. Elsevier Health Sciences:Saunders, 2007.

[41] Regulation (EU) 2018/848 of the European Parliament and of the Council of 30 May 2018 on organic production and labelling of organic products and repealing Council Regulation (EC) No 834/2007.

[42] Spears JW, Harvey RW, Segerson EC. Effects of marginal selenium deficiency and winter protein supplementation on growth, reproduction and selenium status of beef cattle. J Anim Sci. 1986;63(2): 586–594. 10.2527/jas1986.632586x 3759693

[43] Ebrahimi M, Towhidi A, Ganjkhanlou M, Amini M. The effects of organic selenium (Sel-Plex) on viability of pneumonic Holstein sickling calves. Int J Vet Res. 2011;5(3): 163–168. 10.22059/IJVM.2011.23843

[44] Wichtel JJ, Craigie AL, Freeman DA, Varela-Alvarez H, Williamson NB. Effect of selenium and iodine supplementation on growth rate and on thyroid and somatotropic function in dairy calves at pasture. J Dairy Sci. 1996;79(10): 1865–1872. 10.3168/jds.s0022-0302(96)76554-2 8923257

[45] Castellan DM, Maas JP, Gardner IA, Oltjen JW, Sween ML. Growth of suckling beef calves in response to parenteral administration of selenium and the effect of dietary protein provided to their dams. J Am Vet Med Assoc. 1999;214: 816–821. 10101414

[46] Hidiroglou M, Jenkins KJ. Effects of selenium and vitamin E, and copper administrations on weight gains of beef cattle raised in selenium-deficient area. Can J Anim Sci. 1975;55: 307–313.

[47] Jones DG, Stevenson L. Influence of subclinical excesses of selenium and/or vitamin E on clinical biochemistry and antibody responses in mice. J Nutr Immun. 1993;2(1): 55–70. 10.1300/J053v02n01_07

[48] Nair MP, Schwartz SA. Immunoregulation of natural and lymphokine-activated killer cells by selenium. Immunopharmacol. 1990;19(3): 177–183. doi: 0162-3109(90)90067-O 10.1016/0162-3109(90)90067-o 2394580

[49] Juniper DT, Phipps RH, Jones AK, Bertin G. Selenium Supplementation of Lactating Dairy Cows: Effect on Selenium Contretration on Blood, Milk, Urine, and Feces. J Dairy Sci. 2006;89(9): 3544–3551. 10.3168/jds.S0022-0302(06)72394-3 16899690

[50] Machado VS, Oikonomou G, Lima SF, Bicalho MLS, Kacar C, Foditsch C, et. al The effect of injectable trace minerals (selenium, copper, zinc, and manganese) on peripheral blood leukocyte activity and serum superoxide dismutase activity of lactating Holstein cows. Vet J. 2014;200: 299–304. 10.1016/j.tvjl.2014.02.026 24685102

[51] Weiss WP, Hogan JS. Effect of Selenium Source on Selenium Status, Neutrophil Function, and Response to Intramammary Endotoxin Challenge of Dairy Cows J Dairy Sci. 2005;88: 4366–4374. 10.3168/jds.S0022-0302(05)73123-4 16291628

[52] Dai X, Stanilka JM, Rowe CA, Esteves EA, Nieves C, Spaiser SJ, et al Consuming Lentinula edodes (Shiitake) Mushrooms Daily Improves Human Immunity: A Randomized Dietary Intervention in Healthy Young Adults. J Am Coll Nutr. 2015;34: 478–487. 10.1080/07315724.2014.950391 25866155

[53] Lee SH, Lillehoj HS, Hong YH, Jang SI, Lillehoj EP, Ionescu C, et. al In vitro effects of plant and mushroom extracts on immunological function of chicken lymphocytes and macrophages. Br Polultr Sci. 2010;51: 213–221. 10.1080/00071661003745844 20461582

[54] Kim SP, Park SO, Lee SJ, Nam SH, Friedman M. A Polysaccharide Isolated from the Liquid Culture of Lentinus edodes (Shiitake) Mushroom Mycelia Containing Black Rice Bran Protects Mice against a Salmonella Lipopolysaccharide-Induced Endotoxemia. J Agric Food Chem. 2013;61: 10987 10.1021/jf403173k 24200110

[55] Yu S, Weaver V, Martin K, Cantorna MT. The effects of whole mushrooms during inflammation. BMC Immunol 2009;10: 12 10.1186/1471-2172-10-12 19232107 PMC2649035

