# Supplementary material for: Preliminary study on Se-enriched Lentinula edodes mycelium as a proposal of new feed additive in selenium deficiency
Source: PLoS One. 2020 May 21;15(5):e0233456. doi: 10.1371/journal.pone.0233456 (PMC7241721; doi:10.1371/journal.pone.0233456)
Supplement: S1 Table — (DOCX) [file pone.0233456.s001.docx]

| White blood cells |  |  |  |  |  |  |  |  |
| --- | --- | --- | --- | --- | --- | --- | --- | --- |
| week of study | 0 | 1 | 2 | 3 | 4 | 5 | 6 | 7 |
| 4 | 7.00 | 4.60 | 4.60 | 4.90 | 3.40 | 3.80 | 4.40 | 4.70 |
| 5 | 7.10 | 5.40 | 4.20 | 2.10 | 5.10 | 4.80 | 5.10 | 4.60 |
| 6 | 6.00 | 6.30 | 5.00 | 3.70 | 3.40 | 5.80 | 6.00 | 6.30 |
| mean experimental | 6.70 | 5.43 | 4.60 | 3.57 | 3.97 | 4.80 | 5.17 | 5.20 |
| OD | 0.61 | 0.85 | 0.40 | 1.40 | 0.98 | 1.00 | 0.80 | 0.95 |
| 1 | 6.40 | 8.20 | 4.40 | 5.80 | 5.00 | 3.90 | 3.80 | 4.90 |
| 2 | 4.80 | 5.70 | 5.80 | 7.60 | 4.20 | 4.70 | 5.80 | 3.90 |
| 3 | 6.00 | 5.50 | 7.00 | 4.90 | 5.00 | 5.80 | 4.80 | 5.80 |
| mean control | 5.73 | 6.47 | 5.73 | 6.10 | 4.73 | 4.80 | 4.80 | 4.87 |
| OD | 0.83 | 1.50 | 1.30 | 1.37 | 0.46 | 0.95 | 1.00 | 0.95 |
| Lymphocytes |  |  |  |  |  |  |  |  |
| week of study | 0 | 1 | 2 | 3 | 4 | 5 | 6 | 7 |
| 4 | 3.70 | 3.60 | 3.70 | 4.20 | 3.10 | 3.30 | 3.70 | 4.10 |
| 5 | 2.90 | 3.80 | 3.10 | 1.40 | 3.10 | 3.30 | 3.10 | 3.30 |
| 6 | 3.40 | 3.70 | 3.00 | 2.20 | 2.40 | 3.00 | 3.50 | 3.70 |
| mean experimental | 3.33 | 3.70 | 3.27 | 2.60 | 2.87 | 3.20 | 3.43 | 3.70 |
| OD | 0.40 | 0.10 | 0.38 | 1.44 | 0.40 | 0.17 | 0.31 | 0.40 |
| 1 | 4.80 | 3.30 | 3.40 | 4.00 | 3.80 | 3.20 | 2.90 | 4.00 |
| 2 | 3.30 | 3.90 | 4.30 | 4.80 | 3.30 | 3.70 | 4.00 | 3.00 |
| 3 | 4.10 | 4.20 | 4.60 | 3.40 | 3.80 | 4.00 | 3.50 | 4.00 |
| mean control | 4.07 | 3.80 | 4.10 | 4.07 | 3.63 | 3.63 | 3.47 | 3.67 |
| OD | 0.75 | 0.46 | 0.62 | 0.70 | 0.29 | 0.40 | 0.55 | 0.58 |
| Monocytes |  |  |  |  |  |  |  |  |
| week of study | 0 | 1 | 2 | 3 | 4 | 5 | 6 | 7 |
| 4 | 0.80 | 0.30 | 0.40 | 0.30 | 0.20 | 0.30 | 0.30 | 0.20 |
| 5 | 0.80 | 0.60 | 0.30 | 0.30 | 0.50 | 0.50 | 0.50 | 0.50 |
| 6 | 0.60 | 0.50 | 0.5 | 0.50 | 0.40 | 0.50 | 0.60 | 0.70 |
| mean experimental | 0.73 | 0.47 | 0.35 | 0.37 | 0.37 | 0.43 | 0.47 | 0.47 |
| OD | 0.12 | 0.15 | 0.07 | 0.12 | 0.15 | 0.12 | 0.15 | 0.25 |
| 1 | 0.60 | 0.90 | 0.40 | 0.60 | 0.40 | 0.30 | 0.30 | 0.50 |
| 2 | 0.60 | 0.60 | 0.50 | 0.70 | 0.40 | 0.40 | 0.60 | 0.30 |
| 3 | 0.90 | 0.50 | 0.70 | 0.50 | 0.40 | 0.60 | 0.50 | 0.60 |
| mean control | 0.70 | 0.67 | 0.53 | 0.60 | 0.40 | 0.43 | 0.47 | 0.47 |
| OD | 0.17 | 0.21 | 0.15 | 0.10 | 0.00 | 0.15 | 0.15 | 0.15 |
| Granulocytes |  |  |  |  |  |  |  |  |
| week of study | 0 | 1 | 2 | 3 | 4 | 5 | 6 | 7 |
| 4 | 1.60 | 0.70 | 0.50 | 0.40 | 0.30 | 0.20 | 0.40 | 0.40 |
| 5 | 1.90 | 1.00 | 0.80 | 0.40 | 1.50 | 1.00 | 1.50 | 0.80 |
| 6 | 2.50 | 2.10 | 1.50 | 1.00 | 0.60 | 2.30 | 1.90 | 1.90 |
| mean experimental | 2.00 | 1.27 | 0.93 | 0.60 | 0.80 | 1.17 | 1.27 | 1.03 |
| OD | 0.46 | 0.74 | 0.51 | 0.35 | 0.62 | 1.06 | 0.78 | 0.78 |
| 1 | 1.00 | 4.00 | 0.60 | 1.20 | 0.80 | 0.40 | 0.60 | 0.90 |
| 2 | 0.90 | 1.20 | 1.00 | 2.10 | 0.50 | 0.60 | 1.20 | 0.60 |
| 3 | 1.00 | 0.80 | 1.70 | 1.00 | 0.80 | 1.20 | 0.90 | 1.20 |
| mean control | 0.97 | 2.00 | 1.10 | 1.43 | 0.70 | 0.73 | 0.90 | 0.90 |
| OD | 0.06 | 1.74 | 0.56 | 0.59 | 0.17 | 0.42 | 0.30 | 0.30 |
|  |  |  |  |  |  |  |  |  |
| CD 2+ |  |  |  |  |  |  |  |  |
| week of study | 0 | 1 | 2 | 3 | 4 | 5 | 6 | 7 |
| 4 | 42.00 | 41.40 | 37.10 | 38.90 | 49.10 | 44.80 | 46.30 | 41.10 |
| 5 | 62.90 | 57.90 | 49.30 | 39.80 | 62.50 | 61.20 | 52.00 | 54.60 |
| 6 | 46.50 | 34.60 | 27.30 | 36.90 | 24.60 | 24.40 | 27.50 | 23.90 |
| mean experimental | 50.47 | 44.63 | 37.90 | 38.53 | 45.40 | 43.47 | 41.93 | 39.87 |
| OD | 11.00 | 11.98 | 11.02 | 1.48 | 19.22 | 18.44 | 12.82 | 15.39 |
| 1 | 53.40 | 57.50 | 57.60 | 55.70 | 49.50 | 48.80 | 47.90 | 52.50 |
| 2 | 49.80 | 43.90 | 43.60 | 40.40 | 43.10 | 48.90 | 45.00 | 61.50 |
| 3 | 44.20 | 53.50 | 44.80 | 48.80 | 55.70 | 55.70 | 55.70 | 55.70 |
| mean control | 49.13 | 51.63 | 48.67 | 48.30 | 46.30 | 48.85 | 49.53 | 57.00 |
| OD | 4.64 | 6.99 | 7.76 | 7.66 | 6.30 | 3.96 | 5.53 | 4.56 |
| CD 4+ |  |  |  |  |  |  |  |  |
| week of study | 0 | 1 | 2 | 3 | 4 | 5 | 6 | 7 |
| 4 | 27.30 | 20.70 | 21.70 | 25.00 | 19.10 | 23.80 | 22.90 | 23.80 |
| 5 | 42.20 | 40.70 | 34.90 | 22.60 | 47.30 | 44.60 | 37.00 | 37.90 |
| 6 | 27.30 | 17.60 | 18.20 | 17.30 | 12.60 | 13.70 | 14.40 | 15.80 |
| mean experimental | 32.27 | 26.33 | 24.93 | 21.63 | 26.33 | 27.37 | 24.77 | 25.83 |
| OD | 8.60 | 12.54 | 8.81 | 3.94 | 18.45 | 15.76 | 11.42 | 11.19 |
| 1 | 27.90 | 28.00 | 30.20 | 32.90 | 30.80 | 31.30 | 29.70 | 34.50 |
| 2 | 22.20 | 22.10 | 24.20 | 26.70 | 31.30 | 31.50 | 18.40 | 37.30 |
| 3 | 28.80 | 28.00 | 28.40 | 30.70 | 32.90 | 32.90 | 32.90 | 32.90 |
| mean control | 26.30 | 26.03 | 27.60 | 30.10 | 31.05 | 31.40 | 27.00 | 35.90 |
| OD | 3.58 | 3.41 | 3.08 | 3.14 | 1.10 | 0.87 | 7.62 | 2.23 |
| CD 8+ |  |  |  |  |  |  |  |  |
| week of study | 0 | 1 | 2 | 3 | 4 | 5 | 6 | 7 |
| 4 | 14.70 | 12.10 | 9.89 | 13.20 | 16.50 | 10.40 | 15.90 | 13.70 |
| 5 | 20.30 | 10.70 | 6.18 | 11.50 | 14.80 | 13.30 | 14.10 | 15.20 |
| 6 | 14.30 | 12.90 | 7.48 | 10.70 | 12.00 | 10.30 | 9.85 | 14.50 |
| mean experimental | 16.43 | 11.90 | 7.85 | 11.80 | 14.43 | 11.33 | 13.28 | 14.47 |
| OD | 3.35 | 1.11 | 1.88 | 1.28 | 2.27 | 1.70 | 3.11 | 0.75 |
| 1 | 22.90 | 16.60 | 15.80 | 17.00 | 14.20 | 12.60 | 16.10 | 12.90 |
| 2 | 18.10 | 13.70 | 12.90 | 15.40 | 15.70 | 16.00 | 20.60 | 18.80 |
| 3 | 10.50 | 17.70 | 15.20 | 14.20 | 17.00 | 17.00 | 17.00 | 17.00 |
| mean control | 17.17 | 16.00 | 14.63 | 15.53 | 14.95 | 14.30 | 17.90 | 15.85 |
| OD | 6.25 | 2.07 | 1.53 | 1.40 | 1.40 | 2.31 | 2.38 | 3.02 |
| WC 4+ |  |  |  |  |  |  |  |  |
| week of study | 0 | 1 | 2 | 3 | 4 | 5 | 6 | 7 |
| 4 | 11.30 | 13.60 | 12.20 | 12.20 | 10.90 | 11.70 | 12.30 | 11.80 |
| 5 | 9.02 | 11.70 | 11.30 | 11.30 | 12.90 | 14.50 | 11.70 | 13.80 |
| 6 | 14.40 | 13.50 | 15.70 | 15.70 | 10.20 | 11.30 | 14.40 | 12.70 |
| mean experimental | 11.57 | 12.93 | 13.07 | 13.07 | 11.33 | 12.50 | 12.80 | 12.77 |
| OD | 2.70 | 1.07 | 2.32 | 2.32 | 1.40 | 1.74 | 1.42 | 1.00 |
| 1 | 10.60 | 10.40 | 15.00 | 15.00 | 11.20 | 12.80 | 14.40 | 12.20 |
| 2 | 14.50 | 17.10 | 12.20 | 12.20 | 15.30 | 11.90 | 12.40 | 12.30 |
| 3 | 16.60 | 16.80 | 16.80 | 16.80 | 13.25 | 12.80 | 14.40 | 12.30 |
| mean control | 13.90 | 14.77 | 14.67 | 14.67 | 13.25 | 12.50 | 13.73 | 12.27 |
| OD | 3.04 | 3.78 | 2.32 | 2.32 | 2.05 | 0.52 | 1.15 | 0.06 |
